# Contrasted enzymatic cocktails reveal the importance of cellulases and hemicellulases activity ratios for the hydrolysis of cellulose in presence of xylans

**DOI:** 10.1186/s13568-016-0196-x

**Published:** 2016-03-22

**Authors:** Eve Dondelinger, Nathalie Aubry, Fadhel Ben Chaabane, Céline Cohen, Jean Tayeb, Caroline Rémond

**Affiliations:** Université de Reims Champagne-Ardenne, UMR614 Fractionnement des AgroRessources et Environnement, 51100 Reims, France; INRA, UMR614 Fractionnement des AgroRessources et Environnement, 51100 Reims, France; IFP Energies nouvelles, 1 et 4 Avenue de Bois-Préau, 92852 Rueil-Malmaison, France

**Keywords:** Cellulase, β-Glucosidase, Xylanase, β-xylosidase, Ethanol, Biorefinery

## Abstract

Various enzymatic cocktails were produced from two *Trichoderma reesei* strains, a cellulase hyperproducer strain and a strain with β-glucosidase activity overexpression. By using various carbon sources (lactose, glucose, xylose, hemicellulosic hydrolysate) for strains growth, contrasted enzymatic activities were obtained. The enzymatic cocktails presented various levels of efficiency for the hydrolysis of cellulose Avicel into glucose, in presence of xylans, or not. These latter were also hydrolyzed with different extents according to cocktails. The most efficient cocktails (TR1 and TR3) on Avicel were richer in filter paper activity (FPU) and presented a low ratio FPU/β-glucosidase activity. Cocktails TR2 and TR5 which were produced on the higher amount of hemicellulosic hydrolysate, possess both high xylanase and β-xylosidase activities, and were the most efficient for xylans hydrolysis. When hydrolysis of Avicel was conducted in presence of xylans, a decrease of glucose release occurred for all cocktails compared to hydrolysis of Avicel alone. Mixing TR1 and TR5 cocktails with two different ratios of proteins (1/1 and 1/4) resulted in a gain of efficiency for glucose release during hydrolysis of Avicel in presence of xylans compared to TR5 alone. Our results demonstrate the importance of combining hemicellulase and cellulase activities to improve the yields of glucose release from Avicel in presence of xylans. In this context, strategies involving enzymes production with carbon sources comprising mixed C5 and C6 sugars or combining different cocktails produced on C5 or on C6 sugars are of interest for processes developed in the context of lignocellulosic biorefinery.

## Introduction

A challenge for producing glucose from enzymatic hydrolysis of lignocellulosic biomass while limiting the process cost is to perform enzymatic hydrolysis at high solid concentration and to use low enzyme loadings. In this context, the development of optimized enzyme mixtures is of interest. Enzymatic cocktails have to be adapted to lignocellulosic biomass as well as to pretreatment technology which impact largely chemical composition of biomass to hydrolyse. Depending on lignocellulosic biomass and on the severity of the pretreatment technology, xylose and xylo-oligosaccharides (XOs) are released to various extents during the pretreatment step (Chandra et al. 2007). Different approaches can be developed to improve enzymatic fractionation of lignocellulose. Pretreatment of wheat straw at high solid loading (20 % DM) in presence of xylanase conducted to an increase of glucan hydrolysis by Celluclast 1.5 L by a factor 2.1 (Remond et al. 2010). Supplementation of cellulosic cocktails with hemicellulases allowed improving hydrolysis yields of cellulose and hemicelluloses: while increasing the hydrolysis of cellulose and xylan from steam-explosed corn stover, the supplementation by GH11 xylanases of commercial cellulase (Celluclast 1.5 L) allows reducing the cellulase loading by a factor 7 (Hu et al. 2011). This was attributed to the removal of xylans and to the increase of cellulose accessibility by enhancing fiber porosity and swelling (Hu et al. 2011). Another strategy is to develop enzymatic cocktails possessing both cellulases and hemicellulases activities by growing micro-organisms onto various simple or complex substrates. In this way, improved cellulases cocktails contain high hemicellulases level. Recently, *Trichoderma reesei* was cultivated in presence of various commercial sugars to evaluate the impact of these carbon sources onto enzymes produced (Jourdier et al. 2013). In presence of high xylose concentration, cellulases activities (endoglucanase, cellobiohydrolase and β-glucosidase) decreased whereas xylanase activity was more important compared to culture without xylose (Jourdier et al. 2013). Even if significant progress to obtain efficient enzymatic cocktails has already been achieved, their improvement remains a challenge in case of lignocellulosic biomass hydrolysis. To the best of our knowledge, no study concerns the production of enzymes by *T. reesei* growing on hemicellulolytic hydrolysates.

The strategy developed in the present study was based on the use of enzymatic cocktails obtained from *T. reesei*. In this context, various enzymatic cocktails were prepared from *T. reesei* after cultivation with different sugars sources and ratios. This allowed obtaining cocktails containing various levels of cellulases and hemicellulases activities. These cocktails were tested for hydrolysis of Avicel. The effect of the presence of added xylans during the hydrolysis of Avicel was also investigated. Experiments were conducted at high substrate loading and with low enzymes loading in order to mimic the conditions of an industrial process.

The objective of this work was to highlight the impact of the cellulase and some hemicellulases activities in various contrasted enzymatic cocktails during hydrolysis of Avicel, of xylans, and simultaneous hydrolysis of both substrates. Furthermore, combinations of different enzymatic cocktails were evaluated in order to improve the global efficiency of cocktails for cellulose hydrolysis. All cocktails tested in our study were complete cocktails produced by *T. reesei* onto various carbon sources more or less enriched in C5 and C6 sugars.

## Materials and methods

### Materials

Microcrystalline cellulose (Avicel PH-101) and xylose were purchased from Sigma–Aldrich^®^ (St Louis, MO, USA), cellulose content >97 %. Beechwood xylan was supplied by Carl Roth^®^ (Karlsruhe, Germany). XOs were obtained from Cascade Analytical Reagents & Biochemicals (Corvallis, Oregon, USA). XOs contained DP2–5 oligosaccharides.

### Enzymes production in bioreactors

Two strains from *T. reesei* (CL847 and TR3002) were used to produce the different enzymatic cocktails used in this study. *Trichoderma reesei* CL847 is a cellulase hyperproducer strain obtained from NG14 Rut-C30 strain by several steps of mutagenesis and selection, from Cayla Company, Toulouse, France (Portnoy et al. 2011). The strain TR3002 was obtained from the CL847 after introduction of an improved β-glucosidase gene (Ayrinhac et al. 2011). Spores were conserved in cryotubes at −80 °C with 50 % glycerol.

The protocol used to produce the enzymatic cocktails consists in two phases as described previously (Jourdier et al. 2013). Bioreactor cultivations were carried out in Dasgip fedbatch-pro bioreactors with an initial working volume of 750 mL. For each bioreactor, a preculture was performed in a Fernbach flask with 250 mL flask medium culture, inoculated with 10^6^ spores, incubated 72 h at 150 rpm and 30 °C in an Infors rotary shaker. Seventy-five milli-liters were then used to inoculate the bioreactor. Growth phase in batch was performed on 15 g/L glucose at pH 4.8 and 27 °C for 24 h. Then fed-batch was performed at pH 4.0 and 25 °C with feeding at 2 mL/h by a 250 g/L mixed sugars solution (Table 1). The pH was automatically adjusted with 5.5 N NH_3_ solution. Aeration rate was fixed at 30 sL/h and agitation was regulated to maintain at least 40 % dissolved oxygen of its saturation.

**Table 1:** Sugar composition used during the fed-batch mode. C5 refers to hemicellulosic hydrolysate

Experiment	Carbon source	Strain
TR1	20 % xylose/25 % lactose/55 % glucose	TR3002
TR2	100 % C5	CL847
TR3	10 % C5/25 % Lactose/65 % glucose	TR3002
TR4	100 % Lactose	CL847
TR5	75 % C5/25 % Lactose	TR3002

The experiments for TR2 and TR4 production were performed with the strain CL847 while the experiments concerning TR1, TR3 and TR5 were performed with the strain TR3002. The enzymatic cocktails were chosen in order to have contrasted enzymatic activities.

Hemicellulosic hydrolysates referred to pentose (C5) extracts obtained after steam explosion of wheat straw under acidic conditions (H_2_SO_4_ presoaking), followed by washing with water and further concentration by evaporation as described by Warzywoda et al. (1992). The analytical composition of the hemicellulosic hydrolyzate used in this study was: 174 g/L of xylose, 22.5 g/L of arabinose, 27.5 g/L of glucose, 21 g/L of oligomers.

For the preculture before bioreactor cultivations, the medium composition was: cornsteep solid 1.5 g/L; dipotassium phtalate 6 g/L; H_3_PO_4_ 85 % 0.8 mL/L; (NH_4_)_2_SO_4_ 4.2 g/L; MgSO_4_,7H_2_O 0.3 g/L; CaCl_2_,2H_2_O 0.15 g/L; FeSO_4_–7H_2_O 30 mg/L; MnSO_4_,H_2_O 6 mg/L; ZnSO_4_,7H_2_O 8 mg/L; CoNO_3_,6H_2_O 9 mg/L; H_3_BO_3_ 1 mg/L. pH was adjusted to 6.0 with NaOH 30 %.

For bioreactor cultivations, the medium composition was: cornsteep solid 1.5 g/L; KOH 1.66 g/L; H_3_PO_4_ 85 % 2.5 mL/L; (NH_4_)_2_SO_4_ 2.8 g/L; MgSO_4_,7H_2_O 0.6 g/L; CaCl_2_,2H_2_O 0.6 g/L; FeSO_4_–7H_2_O 60 mg/L; MnSO_4_,H_2_O 12 mg/L; ZnSO_4_,7H_2_O 16 mg/L; CoNO_3_,6H_2_O 18 mg/L; H_3_BO_3_ 2 mg/L. pH was adjusted to 4.8 with NH_3_ 20 %.

### Enzyme assays

β-xylosidase and β-glucosidase activities were determined by incubating 0.1 mL of enzymatic cocktail with 0.9 mL *ρ*-nitrophenyl-β-d-xyloside or *ρ*-nitrophenyl-β-d-glucoside as substrates at 5 mM. Reactions were performed during 10 min in 50 mM citrate phosphate buffer, pH 4.8 with appropriate dilute enzyme solutions at 50 °C. Release of *ρ*-nitrophenol (ρNP) was measured by continuous monitoring at 401 nm. One unit of β-xylosidase or β-glucosidase activities was defined as the amount of enzyme releasing 1 µmol of ρNP per minute using the defined conditions.

Endo-β-1,4-xylanase activity was determined by measuring the reducing sugars liberated from beechwood xylan as previously described (Rakotoarivonina et al. 2012). Reaction mixture contained 0.9 mL 0.5 % xylan (w/v) in 50 mM citrate phosphate buffer pH 4.8 and 0.1 mL enzyme solution. Reactions were conducted at 50 °C for 10 min. One unit (IU) was defined as the quantity of enzyme required to liberate 1 µmol of xylose equivalent per minute at 50 °C.

Filter paper activity (FPU) describing the global cellulolytic activity was assayed according to the IUPAC standard Filter Paper Assay (Ghose 1987). The amount of released sugars was quantified from filter paper strip (Whatman no.1, 1 × 6 cm) and reducing sugars were estimated by the DNS method (Miller 1959). One unit of enzyme activity corresponds to the amount of enzyme required to release 1 µmol of glucose equivalent per minute under the assay conditions.

Proteins were measured with the Lowry method (Lowry et al. 1951). Prior to quantification, samples were washed with 10 % trichloroacetic acid during 30 min at 4 °C. Supernatants were thrown after 5 min of centrifugation at 13,000 rpm. Pellets were dried 5 min in a speed-vac and the precipitates were dissolved with 0.08 % sodium hydroxide and 0.4 % sodium carbonate. Proteins concentration was measured in supernatants against BSA standards (0–500 µg/mL).

### Enzymatic hydrolysis

Hydrolysis of 10 % (w/v) Avicel and of 1.5 % (w/v) xylans was performed with enzymes cocktails with a loading of 10 mg proteins/g Avicel or xylans. Reactions were carried out in 50 mM citrate phosphate buffer (pH 4.8) with chloramphenicol (100 ppm) in a thermostatically controlled system Tornado Radleys^®^ (Interchim, Montluçon, France) at 45 °C under agitation at 150 rpm. For some Avicel hydrolysis experiments, 1.5 % (w/v) beechwood xylan, xylose or XOs were added. Experiments conducted with mixtures of cocktails were performed with TR1 supplemented with TR5 with two different ratios of proteins quantities (1/1 and 4/1) with a total protein loading corresponding to 10 mg/g Avicel.

Hydrolysis samples were taken after 24, 48 and 72 h of hydrolysis and were boiled for 10 min to terminate the reaction and stored at −20 °C until carbohydrates analysis. All assays were performed in triplicate.

### Carbohydrates quantification

The glucose concentration was assessed by a glucose oxidase assay with an Analox GL6 glucose analyzer (Imlab, Lille France) and with a standard glucose solution (144 mg/dL, Imlab, Lille France). Quantification of xylose and XOs was performed by HPAEC-PAD (Dionex, Thermo Scientific, Courtaboeuf, France). Before analysis, all samples were filtered (PTFE, 0.22 µm) before injection on a CarboPac PA-1 column (4 × 250 mm, Dionex). Xylose was eluted as previously described (Remond et al. 2010) with fucose as internal standard. XOs (DP2–DP6) were eluted with a 100 mM NaOH and 300 mM sodium acetate gradient with a flow rate of 1 mL/min. Detection was carried out by pulsed amperometry (ED 40, Dionex) and signal sensitivity was increased with a post-column module delivering 300 mM NaOH.

Yields of glucose and xylose released were calculated according to their quantity introduced during the reactions by taking into account their conversion from cellulose and xylans (anhydro correction of 0.9 and 0.88 for glucose and xylose respectively). Yields of XOs released were expressed on the basis of xylose initially present in reaction.

## Results

### Production and characterization of various enzymatic cocktails

Enzymatic cocktails presented various enzymatic activities ratios (Table 2). Cocktail TR1 was the most rich in FPU activity (0.7 FPU/mg) whereas cocktail TR5 contained the lowest FPU activity (0.4 FPU/mg). Cocktails TR3 and TR4 presented similar FPU activity (0.5 FPU/mg) and cocktail TR2 possessed a rather important FPU activity (0.6 FPU/mg). The β-glucosidase activity was the most important in cocktail TR5 (13.1 IU/mg) and decreased by a factor 9 in cocktails TR2 and TR4 (1.4 IU/mg). This activity was rather abundant in cocktail TR3 (10.5 IU/mg) and was intermediary for the cocktail TR1 (6.1 IU/mg). Concerning hemicellulases activities, xylanase and β-xylosidase activities were measured. Cocktails TR2 and TR1 contained high level of xylanase activity (59.1 IU/mg and 53.5 IU/mg respectively). Xylanase activity was lowest for cocktail TR4 (11.9 IU/mg) and was intermediary for cocktails TR3 and TR5 (26.7 and 37.8 IU/mg respectively). Cocktail TR2 which was rich in xylanase activity possessed also high levels of β-xylosidase activity (0.3 IU/mg). However one could observe that cocktail TR1 which presented an important xylanase activity (53.5 IU/mg) did not possess important level of xylosidase activity (0.1 IU/mg). The higher β-xylosidase (0.5 IU/mg) activity was for cocktail TR5 whereas this activity was low for cocktail TR4 (0.03 IU/mg). Finally cocktail TR3 contained intermediary level of β-xylosidase (0.3 IU/mg).

**Table 2:** Proteins concentrations and enzymatic activities (FPU, β-glucosidase, xylanase, β-xylosidase) present in the enzymatic cocktails

	TR1	TR2	TR3	TR4	TR5
Proteins (g/L)	154.0	32.0	58.5	68.0	26.0
FPU (IU/mg)	0.7	0.6	0.5	0.5	0.4
β-Glucosidase (IU/mg)	6.1	1.4	10.5	1.4	13.1
Xylanase (IU/mg)	53.5	59.1	26.7	11.9	37.8
β-Xylosidase (IU/mg)	0.1	0.3	0.3	0.03	0.5

### Enzymatic hydrolysis of Avicel with various contrasted enzymatic cocktails

**Fig. 1:**
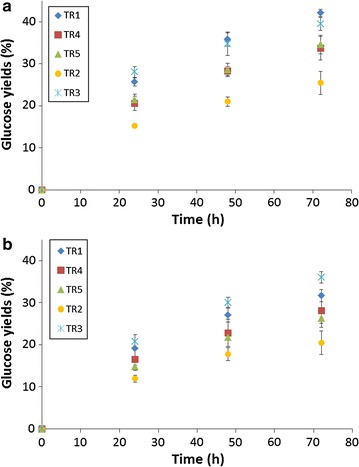
Enzymatic hydrolysis of Avicel 10 % (w/v) by different enzymatic cocktails at 10 mg proteins/g Avicel without xylans (**a**) or in presence of xylans 15 g/L (**b**). Mean values and standard deviations of triplicates are presented

The various enzymatic cocktails were evaluated for their hydrolysis efficiency on cellulose Avicel. In order to reveal subtle differences between all cocktails and to favor low protein concentration use as it should be the case for industrial processes, catalysis was performed with 10 mg proteins/g cellulose. Furthermore, cellulose loading was high as reactions were conducted with 10 % (w/v) of cellulose. The glucose yields are presented in Fig. 1a. For all cocktails, reactions were not finished after 72 h of reaction. This was not surprising as protein concentration was low during hydrolysis experiments. Cocktails TR1 and TR3 were the most efficient for Avicel hydrolysis. Glucose yields were respectively 42.1 ± 0.8 % and 39.5 ± 1.6 % respectively for both these cocktails after 72 h of reaction. Cocktails TR5 and TR4 presented similar kinetic for glucose release which attained respectively 34.6 ± 2.2 % and 33.7 ± 2.2 % after 72 h. Glucose yields were lowest in case of cocktail TR2 and reached 25.5 ± 2.7 % at 72 h.

### Enzymatic hydrolysis of xylans with various contrasted enzymatic cocktails

**Fig. 2:**
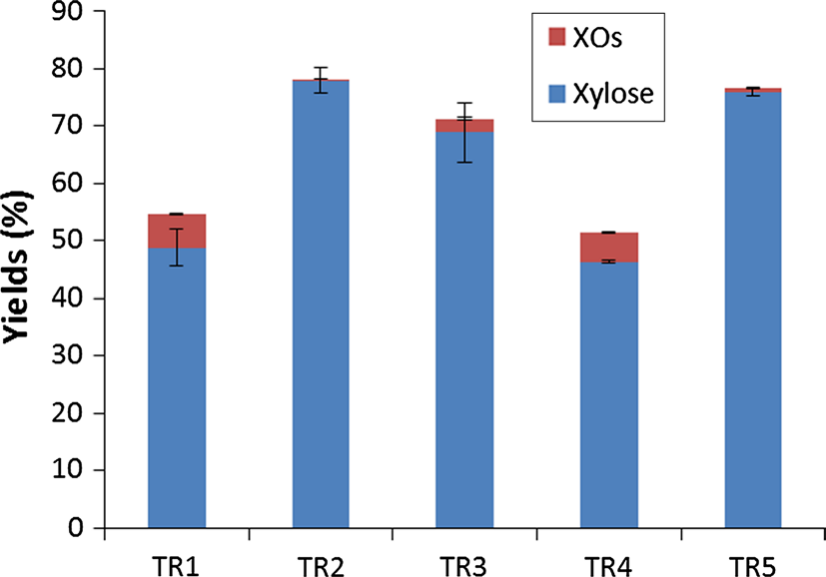
Yields of xylose and xylo-oligosaccharides (XOs) released from xylans (1.5 %, w/v) at 72 h with enzymatic cocktails at 10 mg proteins/g xylans. Mean values and standard deviations of triplicates are presented

Xylan conversion was studied with the various enzymatic cocktails. Yields of xylose and XOs from DP2 to 5 were quantified after 72 h of catalysis (Fig. 2). In case of cocktails TR2 and TR5, xylose release was respectively 77.9 ± 2.2 % and 75.9 ± 0.6 % of total xylose calculated from xylans content. In parallel, XOs content (calculated as % of total xylose from xylans) were low for reactions performed with these both cocktails and reached respectively 0.25 ± 0.05 % and 0.76 ± 0.02 % respectively for TR2 and TR5. Xylose production was less important for cocktail TR3 and reached 68.8 ± 5.1 %. In this case, XOs content represented 2.44 ± 0.15 %. Cocktails TR1 and TR4 were less efficient for xylose release which attained respectively 48.7 ± 3.2 % and 46.3 ± 0.3 % respectively. These both cocktails were also less efficient for XOs hydrolysis as XOs content represented 5.80 ± 0.13 % and 5.13 ± 0.15 % respectively for cocktails TR1 and TR4.

### Enzymatic hydrolysis of Avicel in presence of xylans with various contrasted enzymatic cocktails

Hydrolysis experiments of cellulose were conducted in presence of xylans with the different cocktails. Figure 1b represents the kinetic of glucose released during 72 h of reaction. After 72 h of reactions, yields were 20.5 ± 2.8 %, 26.3 ± 1.2 %, 28.1 ± 2.8 %, 31.7 ± 1.4 % and 36.0 ± 1.4 % respectively for cocktails TR2, TR5, TR4, TR1 and TR3. This classification of increased efficiency displayed according to cocktails was similar to the one obtained for reactions catalyzed without xylans (Fig. 1a). However, an important observation is that for all enzymatic cocktails and during the entire reactions, yields of glucose were lower than those obtained when catalysis was performed in absence of xylans. After 72 h, yields were decreased by factor 1.33 and 1.32 for TR1 and TR5 whereas the decrease was 1.24 and 1.20 for TR2 and TR4. The yield was less affected for cocktail TR3 as it decreased 1.1-fold.

**Fig. 3:**
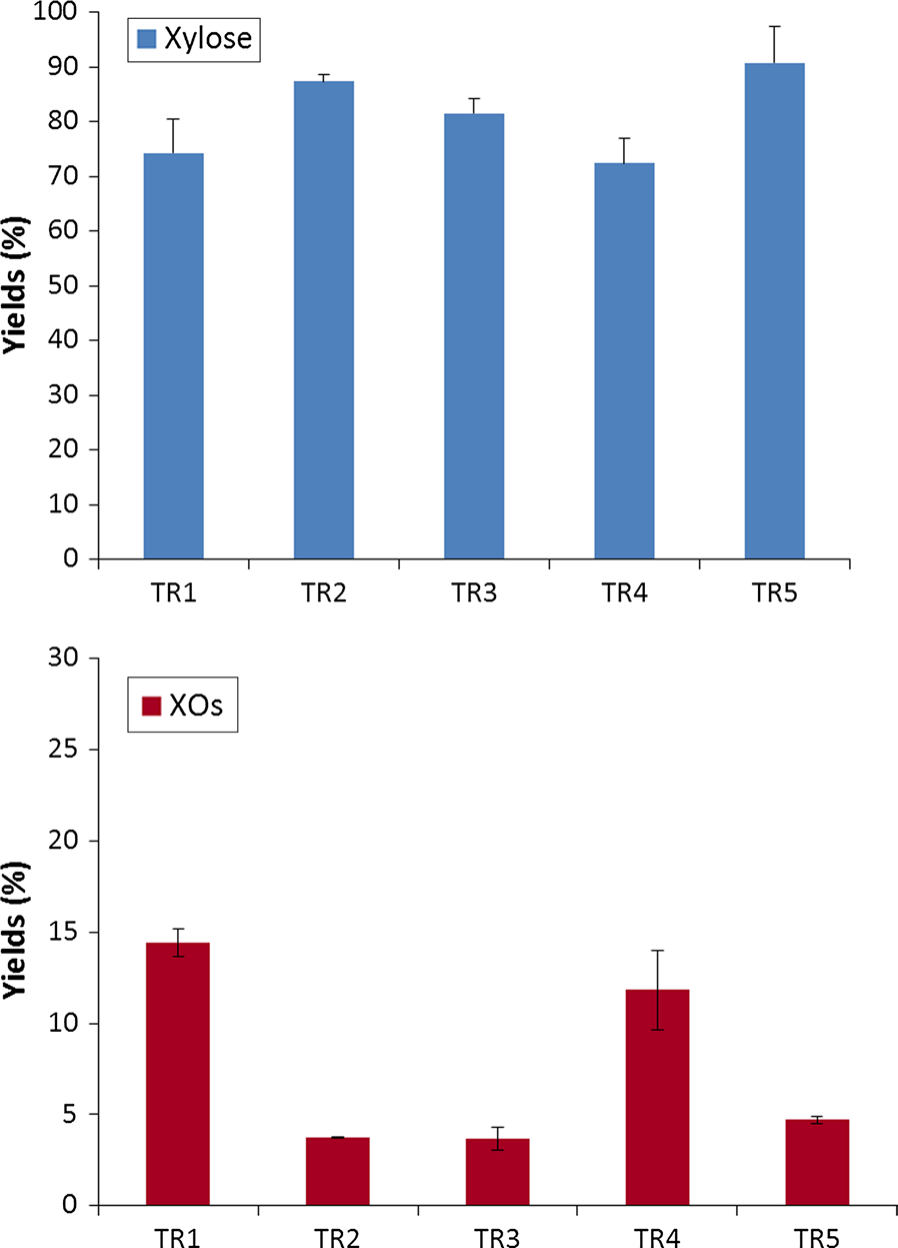
Yields of xylose and xylo-oligosaccharides (XOs) released in presence of cellulose Avicel (10 %, w/v) and xylans (1.5 %, w/v) at 72 h with enzymatic cocktails at 10 mg proteins/g Avicel. Mean values and standard deviations of triplicates are presented

Quantification of xylose and XOs present in solutions at 72 h is presented in Fig. 3. As observed for hydrolysis reactions of xylans, the various enzymatic cocktails did not present the same efficiency for xylans hydrolysis. Cocktails TR5 and TR2 were the most efficient and liberated xylose with yields respectively 90.7 ± 6.7 % and 87.3 ± 1.2 %. XOs concentrations were respectively 4.7 ± 0.2 % and 3.7 ± 0.7 % for TR5 and TR2. Yield of xylose released was respectively 81.5 ± 2.7 %, 74.2 ± 6.3 % and 72.4 ± 4.5 % for cocktails TR3, TR1 and TR4 respectively. XOs concentrations reached 3.7 ± 0.6 %, 11.8 ± 2.2 % and 14.4 ± 0.7 % respectively for TR3, TR4 and TR1. These results are in accordance with the level of xylosidase activity poorly present in TR4 and TR1 cocktails.

**Fig. 4:**
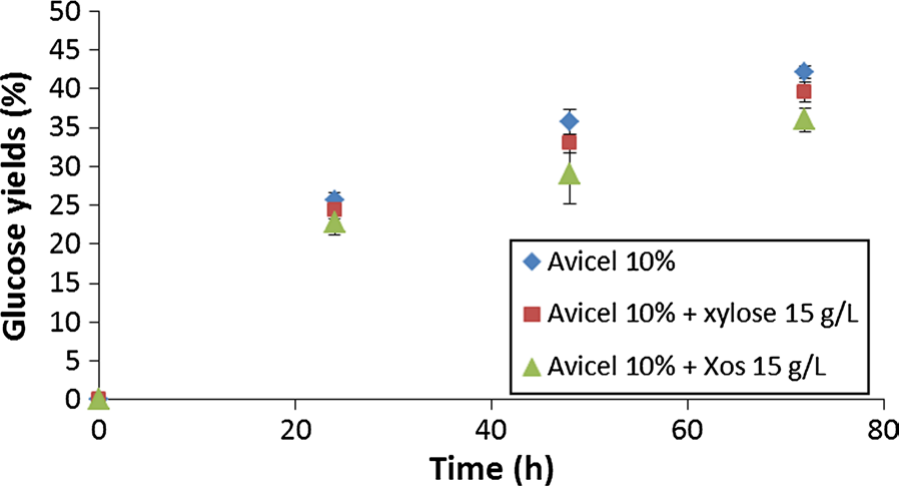
Yields of glucose released from Avicel (10 %, w/v) in absence or in presence of xylose and XOs (DP 2–5) with TR1 cocktail at 10 mg proteins/g Avicel. Mean values and standard deviations of triplicates are presented

In the present study, hydrolysis of Avicel was tested with TR1 cocktail in presence of xylose or in presence of XOs (DP 2–5) (Fig. 4). Whereas xylose did not lead to any significant reduction in glucose yields, presence of XOs from DP2 to 5 induced a lower glucose release from Avicel notably at 48 and 72 h. Glucose yields were respectively 35.8 and 42.1 % after 48 and 72 h without XOs and decreased to 29.1 and 36 % after 48 and 72 h in presence of XOs which represents a decrease by 1.23-fold and 1.17-fold respectively. Similar reduction of glucose release was obtained for other enzymatic cocktails (data not shown).

### Effect of cocktails mixture for hydrolysis efficiency

**Fig. 5:**
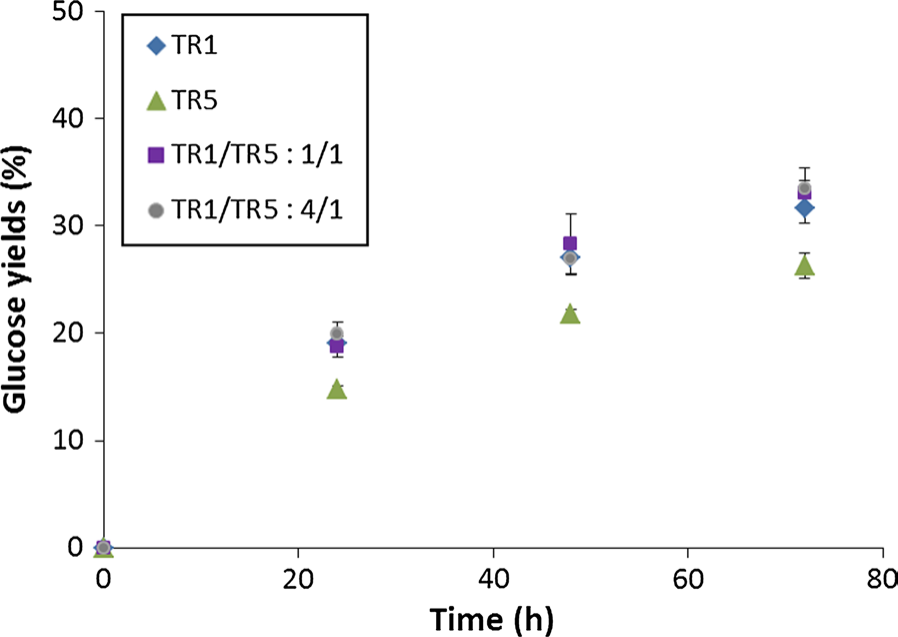
Enzymatic hydrolysis of Avicel 10 % (w/v) in presence of xylans 15 g/L with different enzymatic cocktails and mixtures of cocktails with 10 mg total proteins/g Avicel. Mean values and standard deviations of triplicates are presented

**Fig. 6:**
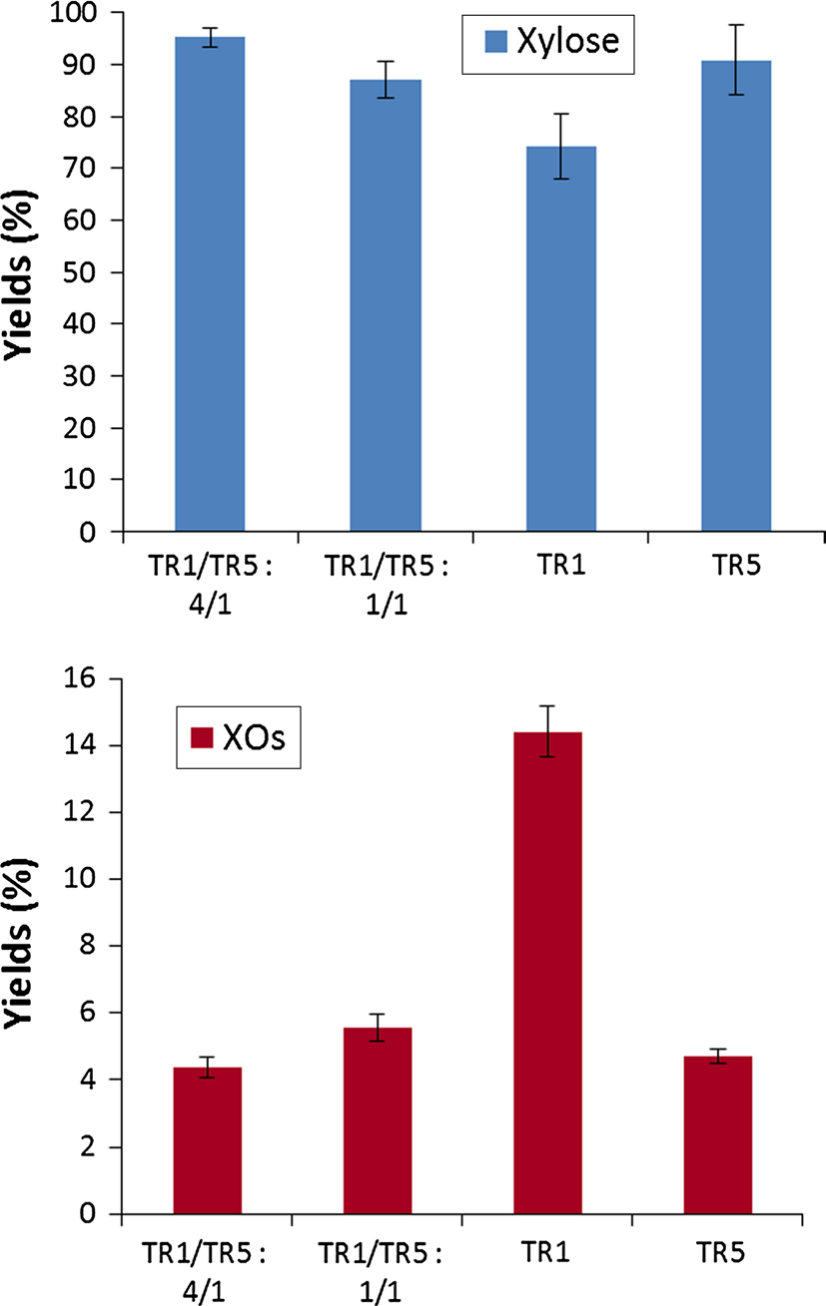
Yields of xylose and xylo-oligosaccharides (XOs) released in presence of cellulose Avicel (10 %, w/v) and xylans (1.5 %, w/v) at 72 h with TR1 and TR5 alone or with mixtures of TR1/TR5. For all conditions, the enzyme loading was 10 mg total proteins/g Avicel. Mean values and standard deviations of triplicates are presented

In order to investigate the impact of enzymatic activities enrichment, experiments were conducted with some cocktails mixtures. The objective was to test their effect on hydrolysis of cellulose in presence of xylans. In that way, mixtures were prepared with TR1 supplemented with TR5 with two different ratios of proteins quantities (1/1 and 4/1) with a total protein loading corresponding to 10 mg/g Avicel (Table 3). These combinations were chosen in order to obtain cocktails presenting high levels of FPU, β-glucosidase, xylanase and β-xylosidase activities. For both ratios tested, mixing TR1 with TR5 induced a decrease of FPU and xylanase activities whereas β-glucosidase and β-xylosidase activities were increased compared to TR1. In contrary, in case of TR5, complementation with TR1 with both ratios induced more important FPU and xylanase activities and less high levels of β-glucosidase and β-xylosidase activities. Hydrolysis experiments were performed with these cocktails mixtures in same conditions as for previous experiments: 10 % Avicel in presence of xylans 15 g/L with a total loading of 10 mg proteins/g Avicel. For both ratios tested, release of glucose reached similar yields (33.1 ± 1.2 % and 33.5 ± 1.9 % respectively for ratios 1/1 and 4/1 after 72 h) as obtained for TR1 alone (31.7 ± 1.4 % after 72 h) (Fig. 5). Compared to TR5 alone (26.3 ± 1.2 %), both mixtures led to increased glucose yields which were 1.27-fold higher at 72 h indicating that the more important cellulase activity in cocktail mixtures could probably be responsible for these higher yields. Xylose and XOs present at 72 h were quantified (Fig. 6). Xylose release was higher for ratio 4/1 (95.2 ± 1.9 %) compared to ratio 1/1 (87 ± 3.4 %). The xylose release is more important with TR1/TR5: 1/4 mixture compared to TR5 alone (90.7 %). XOs were detected with concentrations reaching 4.3 ± 0.3 % and 5.5 ± 0.4 % respectively for TR1/TR5 ratios 1/4 and 1/1.

**Table 3:** Proteins concentrations and enzymatic activities measured from cocktails mixtures (FPU, β-glucosidase, xylanase, β-xylosidase) present in the cocktails mixtures

	TR1/TR5 1/1	TR1/TR5 4/1
Proteins (g/L)	89.4	125.0
FPU (IU/mg)	0.5	0.6
β-Glucosidase (IU/mg)	10.2	7.0
Xylanase (IU/mg)	45.6	50.0
β-Xylosidase (IU/mg)	0.3	0.2

## Discussion

Contrasted enzymatic activities ratios characterizing the obtained cocktails could be related to the strain and to the substrates used during the culture of *T. reesei* for enzymes production. The experiments for TR2 and TR4 production were performed with the strain CL847 while the experiments concerning TR1, TR3 and TR5 were performed with the strain TR3002 which have an improved β-glucosidase expression capacity explaining why β-glucosidase activity is higher in case of these three cocktails. For all the experiments, enzymes production was carried out in carbon-limited fed-batch mode with lactose, hemicellulosic hydrolysate (C5) and mix of lactose, glucose xylose and C5 with different proportions. The hemicellulosic hydrolysate corresponds to the water extracts of steam-exploded biomass. It is mainly composed of monomeric pentoses (xylose, arabinose) and oligomeric pentoses both resulting from the thermo-chemical hydrolysis. Before being used for cellulase biosynthesis, the hemicellulosic hydrolysate was mixed with lactose and eventually glucose in the feeding solution as described previously (Ben Chaabane and Marchal 2013). TR2 corresponds to an experiment where only the hemicellulosic hydrolysate was used as carbon source in the fed-batch solution. TR4 corresponds to an experiment where only the lactose was used in the fed-batch solution. Ten percent of hemicellulosic hydrolysate was used as carbon source during the culture experiment of TR3 and 75 % during the culture experiment of TR5. Finally, the experiment TR1 was carried out using 20 % xylose instead of the hemicellulosic hydrolysate. Results indicate that xylose induces high xylanase activity but lowers β-xylosidase activity compared to hemicellulosic hydrolysates.

Various enzymes, expressed by *T. reesei*, are involved in lignocellulosic biomass fractionation. In a previous study concerning the analysis of the secretome of *T. reesei* CL847 growing on lactose-based media, 22 biomass-degrading enzymes were identified and represented 93 % of the secretome (Herpoel-Gimbert et al. 2008). These enzymes correspond notably to 2 cellobiohydrolases (CBH), 4 endoglucanases, 1 β-glucosidase, 3 xylanases, 1 β-xylosidase, 1 mannanase. Cellulases and hemicellulases production by *T. reesei* is known to be dependent on carbon sources (Juhász et al. 2005). One could suppose that using various C5 and C6 carbon sources led to modulations of secreted enzymes by CL847 and TR3002 strains used in our study. The objective of our study was to relate the enzymatic activities to the yields of cellulose and xylans hydrolysis. In this context, the characterization of enzymatic cocktails was based on the measurement of enzymatic activities (FPU, β-glucosidase, xylanase, β-xylosidase) supposed to play an essential role during cellulose and xylans hydrolysis. As beechwood xylans used for enzymatic hydrolysis experiments contain few arabinose (<1 % DM) and no esterified groups (acetyl, feruloyl), arabinosidase and esterases activities were not quantified.

In regards of enzymatic activities present in the different cocktails, efficiency of cocktails for cellulose hydrolysis could be explained by FPU and β-glucosidase activities levels. High FPU and β-glucosidase activities considered as dissociated do not allow explaining the various glucose yields obtained with the different cocktails. Indeed, TR2 and TR5 cocktails which contain respectively high FPU and β-glucosidase activities are not those generating maximal glucose release. Cocktails TR1 and TR3 giving rise to the most important glucose yields were characterized by high levels of FPU activities as well as by low ratios between FPU and β-glucosidase activities (respectively 0.11 and 0.05). In comparison, lower efficiency of cocktails TR5 and TR4 for cellulose hydrolysis could be explained by a less important FPU activity for TR5 (in spite of a low ratio FPU/β-glucosidase: 0.03) and by a higher ratio FPU/β-glucosidase (0.36) for TR4. In case of cocktail TR2, FPU activity was as important as for cocktail TR4 and higher compared to FPU activity of TR5, however the high ratio FPU/β-glucosidase (0.43) was probably responsible for the limited glucose release. β-Glucosidase activity represents an essential factor for the design of cellulase cocktails. Indeed β-glucosidases are responsible for glucose release from cellobiose produced synergistically by endoglucanases and CBH during cellulose hydrolysis. Furthermore β-glucosidases decrease the accumulation of cellobiose during catalysis and thus limit CBH inhibition by this disaccharide (Holtzapple et al. 1990). In that way, recent commercial cellulase cocktails have been supplemented with β-glucosidase activity.

TR2 and TR5 cocktails, which were the most efficient for xylose production from xylans and for which XOs content were the less abundant, possessed both high xylanase and β-xylosidase activities. Xylanase activity was less important within cocktail TR3 which could probably explain the lower yield of xylans conversion into xylose compared to TR2 and TR5 cocktails. The low β-xylosidase activity level within TR1 and TR4 cocktails could be responsible of the low xylose production observed with these cocktails in spite of a high xylanase activity level for TR1 cocktail.

In comparison with hydrolysis experiments performed on xylans, total yields of xylose release were more important for hydrolysis of xylans in presence of Avicel. This could be explained by a loading of enzymatic proteins 6.6-folds more important for hydrolysis of Avicel in presence of xylans compared to enzymes loading for xylans hydrolysis. Globally, when hydrolysis was conducted simultaneously onto cellulose and xylans, release of glucose was decreased compared to action onto separated Avicel. This indicates a lesser efficiency of cellulases in this case. Recent studies revealed that xylose, XOs and xylans have a negative impact during hydrolysis of cellulose with cellulases. For XOs, their negative impact during cellulose hydrolysis with cellulases was reported in numerous studies (Hu et al. 2013; Shi et al. 2011). XOs inhibitory effect is higher than the one obtained in presence of xylose (Qing et al. 2010). A mixture of XOs from DP7 to 16 was recovered from hydrothermally pretreated wheat straw (Kont et al. 2013) and these oligosaccharides induced an inhibitory effect 100-fold more important on CBH from *T. reesei* than cellobiose. By mimicking the structure of cellulose chain, these oligosaccharides bind the active site of CBHs (Kont et al. 2013). Competitive inhibition seems to be partly responsible of the negative impact of XOs on cellulases efficiency (Qing et al. 2010) notably on CBHI (Zhang and Viikari 2012). Structural resolution of the CBH Cel7A from *Hypocrea jecorina* complexed with XOs indicated that xylotriose, xylotetraose and xylopentaose bind predominantly to the entrance of the substrate-binding tunnel of the enzyme and that an second alternative binding mode occurs near the catalytic center of the enzyme (Momeni et al. 2015). The data obtained during hydrolysis of Avicel in presence of xylans, indicate that a larger proportion of residual cellulose and a lesser extent part of xylans could remain in reactional media. Previous experiments demonstrated that presence of xylans was a factor decreasing cellulases efficiency notably by limiting cellulose accessibility (Penttilä et al. 2013; Zhang et al. 2012; Zhang and Viikari 2014). This could be attributed to the adsorption of xylans chains onto cellulose surface (Kohnke et al. 2008, 2011). One could not exclude that binding of xylans chains into the active site of cellulases occurs leading to their inhibition (Zhang et al. 2012).

Results obtained with cocktails mixtures indicate that TR1/TR5 with both ratios represented improved enzymatic cocktails for glucose release during hydrolysis of Avicel in presence of xylans compared to TR5 alone. Mixture 4/1 was also more effective for xylose release compared to TR5 alone. In comparison to TR1, the efficiency of TR1/TR5 mixtures was most important for xylose release but no gain was obtained for glucose production. In case of complex enzymatic mixtures, as it is the case in our study, correlating yields of products to levels of enzymatic activities is not an easy task. Adding pure enzymes to complex enzymatic cocktails represents a simplest approach. In this way, Gao et al. (2011) tailored optimal enzymatic cocktails including cellulases (endoglucanase, cellobiohydrolase and β-glucanase activities) and hemicellulases (xylanase, β-xylosidase, α-arabinosidase and α-glucuronidase activities) for the hydrolysis of AFEX pretreated corn stover (Gao et al. 2011). This allowed recovering high yields of glucose (80 %) and xylose (70 %) with a reasonable protein loading (20 mg/g glucan). In a same way, on steam exploded wheat straw the supplementation of commercial cellulases with a xylanase and an arabinosidase gave rise to 10 % higher glucose yield (Alvira et al. 2011). Another previous study showed that improvement of enzymatic cocktails largely depends on the substrate used for hydrolysis: the supplementation of a cellulase cocktail with xylanase and β-xylosidase activities improved glucan conversion from corn stover pretreated with AFEX and dilute acid (increase of 27 and 8 % respectively); furthermore the addition of these both hemicellulases gave more benefic impact when adding them several hours before the addition of cellulase compared to a latter addition (Qing and Wyman 2011).

In our study, *T. reesei* strains modify their enzymatic activities levels produced according to the sugar nature present as carbon sources. Hydrolysis of Avicel with the various cocktails was more important when cocktails were rich in FPU activity and when ratio FPU/β-glucosidase was low. The presence of xylans during Avicel hydrolysis impacted negatively the efficiency of cellulases for glucose release. By mixing TR1 and TR5 cocktails, improved yield of Avicel hydrolysis in presence of xylans was obtained demonstrating the importance of combining hemicellulases and cellulasic activities. These results highlight the importance of optimizing the enzymatic activities levels to obtain efficient enzymatic cocktails for complex substrates hydrolysis.

## References

[CR1] Alvira P, Negro MJ, Ballesteros M (2011). Effect of endoxylanase and α-L-arabinofuranosidase supplementation on the enzymatic hydrolysis of steam exploded wheat straw. Bioresour Technol.

[CR2] Ayrinhac C, Margeot A, Lopes Ferreira N, Ben Chaabane F, Monot F, Ravot G, Sonet JM, Fourage L (2011). Improved saccharification of wheat straw for biofuel production using an engineered secretome of *Trichoderma reesei*. Org Process Res Dev.

[CR3] Ben Chaabane F, Marchal R (2013). Upgrading the hemicellulosic fraction of biomass into biofuel. Oil Gas Sci Technol.

[CR4] Chandra RP, Bura R, Mabee WE, Berlin A, Pan X, Saddler JN. Substrate pretreatment: the key to effective enzymatic hydrolysis of lignocellulosics? In: Olsson L, editor. Biofuels. Adv Biochem Eng Biotechnol. 2007;108:67–93. 10.1007/10_2007_064 17530205

[CR5] Gao DH, Uppugundla N, Chundawat SPS, Yu XR, Hermanson S, Gowda K, Brumm P, Mead D, Balan V, Dale BE (2011). Hemicellulases and auxiliary enzymes for improved conversion of lignocellulosic biomass to monosaccharides. Biotechnol Biofuels.

[CR6] Ghose TK (1987). Measurement of cellulase activities. Pure Appl Chem.

[CR7] Herpoel-Gimbert I, Margeot A, Dolla A, Jan G, Molle D, Lignon S, Mathis H, Sigoillot JC, Monot F, Asther M (2008). Comparative secretome analyses of two *Trichoderma reesei* RUT-C30 and CL847 hypersecretory strains. Biotechnol Biofuels.

[CR8] Holtzapple M, Cognata M, Shu Y, Hendrickson C (1990). Inhibition of *Trichoderma reesei* cellulase by sugars and solvents. Biotechnol Bioeng.

[CR9] Hu JG, Arantes V, Pribowo A, Saddler JN (2013). The synergistic action of accessory enzymes enhances the hydrolytic potential of a “cellulase mixture” but is highly substrate specific. Biotechnol Biofuels.

[CR10] Hu JG, Arantes V, Saddler JN (2011). The enhancement of enzymatic hydrolysis of lignocellulosic substrates by the addition of accessory enzymes such as xylanase: is it an additive or synergistic effect?. Biotechnol Biofuels.

[CR11] Jourdier E, Cohen C, Poughon L, Larroche C, Monot F, Ben Chaabane F (2013). Cellulase activity mapping of *Trichoderma reesei* cultivated in sugar mixtures under fed-batch conditions. Biotechnol Biofuels.

[CR12] Juhász T, Szengyel Z, Réczey K, Siika-Aho M, Viikari L (2005). Characterization of cellulases and hemicellulases produced by *Trichoderma reesei* on various carbon sources. Process Biochem.

[CR13] Kohnke T, Pujolras C, Roubroeks JP, Gatenholm P (2008). The effect of barley husk arabinoxylan adsorption on the properties of cellulose fibres. Cellulose.

[CR14] Kohnke T, Ostlund A, Brelid H (2011). Adsorption of arabinoxylan on cellulosic surfaces: influence of degree of substitution and substitution pattern on adsorption characteristics. Biomacromolecules.

[CR15] Kont R, Kurasin M, Teugjas H, Valjamae P (2013). Strong cellulase inhibitors from the hydrothermal pretreatment of wheat straw. Biotechnol Biofuels.

[CR16] Lowry OH, Rosebrough NJ, Farr AL, Randall RJ (1951). Protein measurement with the Folin phenol reagent. J Biol Chem.

[CR17] Miller GL (1959). Use of dinitrosalicylic acid reagent for determination of reducing sugar. Anal Chem.

[CR18] Momeni MH, Ubhayasekera W, Sandgren M, Stahlberg J, Hansson H (2015). Structural insights into the inhibition of cellobiohydrolase Cel7A by xylo-oligosaccharides. FEBS J.

[CR19] Penttilä PA, Várnai A, Pere J, Tammelin T, Salmén L, Siika-aho M, Viikari L, Serimaa R (2013). Xylan as limiting factor in enzymatic hydrolysis of nanocellulose. Bioresour Technol.

[CR20] Portnoy T, Margeot A, Seidl-Seiboth V, Le Crom S, Ben Chaabane F, Linke R, Seiboth B, Kubicek CP (2011). Differential regulation of the cellulase transcription factors XYR1, ACE2, and ACE1 in *Trichoderma reesei* strains producing high and low levels of cellulase. Eukaryot Cell.

[CR21] Qing Q, Wyman CE (2011). Hydrolysis of different chain length xylooliogmers by cellulase and hemicellulase. Bioresour Technol.

[CR22] Qing Q, Yang B, Wyman CE (2010). Xylooligomers are strong inhibitors of cellulose hydrolysis by enzymes. Bioresour Technol.

[CR23] Rakotoarivonina H, Hermant B, Monthe N, Remond C (2012). The hemicellulolytic enzyme arsenal of *Thermobacillus xylanilyticus* depends on the composition of biomass used for growth. Microb Cell Fact.

[CR24] Remond C, Aubry N, Cronier D, Noel S, Martel F, Roge B, Rakotoarivonina H, Debeire P, Chabbert B (2010). Combination of ammonia and xylanase pretreatments: impact on enzymatic xylan and cellulose recovery from wheat straw. Bioresour Technol.

[CR25] Shi J, Ebrik MA, Yang B, Garlock RJ, Balan V, Dale BE, Pallapolu VR, Lee YY, Kim Y, Mosier NS, Ladisch MR, Holtzapple MT, Falls M, Sierra-Ramirez R, Donohoe BS, Vinzant TB, Elander RT, Hames B, Thomas S, Warner RE, Wyman CE (2011). Application of cellulase and hemicellulase to pure xylan, pure cellulose, and switchgrass solids from leading pretreatments. Bioresour Technol.

[CR26] Warzywoda M, Larbre E, Pourquie J (1992). Production and characterization of cellulolytic enzymes from *Trichoderma reesei* grown on various carbon sources. Bioresour Technol.

[CR27] Zhang JH, Viikari L (2012). Xylo-oligosaccharides are competitive inhibitors of cellobiohydrolase I from *Thermoascus aurantiacus*. Bioresour Technol.

[CR28] Zhang JH, Tang M, Viikari L (2012). Xylans inhibit enzymatic hydrolysis of lignocellulosic materials by cellulases. Bioresour Technol.

[CR29] Zhang JH, Viikari L (2014). Impact of xylan on synergistic effects of xylanases and cellulases in enzymatic hydrolysis of lignocelluloses. Appl Biochem Biotech.

